# Hyoid displacement during swallowing function for completely edentulous subjects rehabilitated with mandibular implant retained overdenture

**DOI:** 10.1186/s12903-024-04616-9

**Published:** 2024-08-08

**Authors:** Abdallah Mohammed Ibrahim, Mohamed Elgamal, Elsayed Abdallah Abdel-Khalek

**Affiliations:** 1 Department of Removable prosthodontics, Faculty of Dentistry, Mansoura University, #68 ElGomhoria Street, ElMansoura, P.O.Box: 35516, Eldakahlia, Egypt; 2 Faculty of Dentistry, Horus University, Damietta, Egypt

**Keywords:** Hyoid bone, Denture, Implant, Overdenture, Swallowing

## Abstract

**Background:**

Hyoid bone is attached to the mandible, tongue, larynx, temporal bone, and cervical spine via different types of muscles or ligaments. The tongue, mandible, and hyoid system play a crucial role in swallowing function. This within subject study aimed to evaluate the impact of mandibular implant overdentures on the displacement of the hyoid bones during the swallowing process.

**Methods:**

Twenty five healthy edentulous subjects were selected for participating in the study. New complete dentures were constructed for all the participants. Subsequently two dental implants were inserted in the canine regions of the participant’s mandibular arch. In order to retain the mandibular prosthesis in place, ball attachments were incorporated into the mandibular dentures to convert them into implant overdentures. Using 10 ml of thin liquid bolus, videofluoroscopy swallowing examination was performed in three different oral conditions: without complete dentures (WCD), with complete denture (CDs), and with a mandibular implant overdenture (IODs). ANOVA with Bonferroni test was used to analyze the data in order to determine how the hyoid displacement varied throughout different oral conditions.

**Results:**

Compared to complete dentures, mandibular implant overdentures showed a significant decrease (*P* < 0.05) in both anterior hyoid displacement and duration of hyoid maximum anterior excursion (DOHMAE). However, there was a non-significant difference (*P* > 0.05) between the two oral circumstances in terms of superior hyoid displacement or duration for hyoid maximum elevation (DOHME). There is no penetration or aspiration for both complete denture and implant overdenture oral conditions.

**Conclusion:**

Implant retained overdentures have a positive effect on hyoid displacement during swallowing of thin liquid bolus consistency relative to conventional complete dentures.

**Trial registration:**

Retrospectively registered (NCT06187181) 02/1/2024.

**Supplementary Information:**

The online version contains supplementary material available at 10.1186/s12903-024-04616-9.

## Background

Rehabilitation of edentulous patients is commonly done with conventional complete dentures. However, these dentures often encounter problems with stability and retention, which can reduce the patient’s ability to chew [1]. To address these issues, dental implants have been used to provide a variety of treatment options for restoring completely edentulous arches, including removable prostheses with different attachment systems or fixed dental prostheses [2–4]. Research in this field has shown that conventional complete dentures are no longer the most appropriate first choice for prosthodontic treatment of the edentulous mandible [5]. There is now overwhelming evidence that a two-implant overdenture should be the first choice for treatment [6].

The term dysphagia is used to describe any swallowing dysfunction [7–10] that causes discomfort or impairs the formation of food bolus or transportation of food bolus from the oral cavity to the stomach without entering the airway space [11]. The relation between conventional complete dentures and swallowing function were investigated by several studies [12–14]. One cross-sectional study found a link between complete edentulism and swallowing dysfunction, as well as between wearing dentures and the swallowing process [15].

The swallowing process consists of four phases: the voluntary oral preparatory and oral propulsive phases, and the involuntary pharyngeal and esophageal phases [16]. The physiological swallowing process begins when the hyoid bone moves, which is caused by the contraction of the supra-hyoid muscles and the base of the tongue. The hyoid bone is located in the anterior midline of the neck [17] and can be identified in videofluoroscopy swallowing study (VFSS) images as a relatively radio-opaque horseshoe-shaped structure [18, 19]. The hyoid bone is attached to the mandible, tongue, larynx, temporal bone, sternum, and cervical spine via various muscles and ligaments. The tongue-mandible-hyoid system plays an important role in swallowing function [20, 21]. The superior displacement of the hyoid bone is closely related to epiglottic inversion and closure of the epiglottic vestibule to prevent penetration and aspiration of food bolus during swallowing. The anterior displacement of the hyoid bone is an important factor in opening the upper esophageal sphincter, allowing for clearance of the food bolus into the esophagus [22]. Also, any abnormalities in hyoid bone position can cause pain in the neck, temporal region, and mandible [23].

Videofluoroscopy swallowing study (VFSS) is the ideal and most commonly used method for evaluating swallowing problems. During this evaluation, the subject is asked to swallow food boluses of different sizes and consistencies mixed with a radio-opaque material, while the swallowing process is recorded by fluoroscopy and saved for later analysis. This process allows for visualization of bolus movement and the movement of anatomical structures during swallowing [24, 25].

Previous studies have examined the effect of wearing complete dentures on the extent of anterior and superior hyoid movement during swallowing [12, 13]. They found that swallowing with complete dentures reduce bolus transit time and frequency of laryngeal penetration while the removal of denture expand the pharyngeal area and compensated by increasing the displacement of hyoid bone and larynx. However, there is limited information on the impact of implant overdentures on hyoid displacement during swallowing. Therefore, this research aimed to study the effect of mandibular implant-retained overdentures on anterior and superior hyoid displacement during the swallowing function. The null hypothesis assumes that there will be no significant differences in hyoid bone displacement between implant overdentures and conventional complete dentures.

## Materials and methods

### Patient selection and study design

Twenty five healthy edentulous patients aged from 50 to 70 years participated in this within subject study. All participants were completely edentulous for at least 1 year and seeking complete denture construction at the Outpatient Clinic, Prosthodontics Department, Faculty of Dentistry. Power analysis was conducted using computer software program (G*power, version 3.1.5, Kiel) to determine sample size. The calculations were based on data from previous research [12] which found a significant difference in clinical outcomes between two treatment modalities. The sample size was determined to be 25 participants [Effect size = 0.46, type I error (α) = 0.05, type II error (β) = 0.80]. Before being included in the study, all participants were screened for any systemic diseases that could potentially affect the osseointegration of the dental implants. They were also required to have a class I maxillomandibular relationship and no history of swallowing problems. Any participants had skeletal deformities, sleep apnea, smoking, head and neck surgery, or temporomandibular joint disorders were excluded from the study. The study was approved by the Faculty of Dentistry’s ethical committee (NO. A19061222) and written consent was obtained from each participant after a thorough explanation of the study’s purpose, methods, benefits, and risks.

### Prosthetic and surgical procedures

For every participant a newly constructed complete denture with lingualized balanced occlusal scheme and optimal flange extension was delivered. A clear acrylic resin copy of the mandibular denture had multiple small holes filled with radiopaque gutta-percha was used to create the radiographic stent. Using a dual scan method, cone beam computerized tomography [i-CAT] was utilized to create the stereolithographic surgical guide. A guided surgical protocol was used to place 2 dental implants (Dentium implants, 12–14 mm in length, 3.6–4 mm in diameter, Superline II) in the canine areas of the mandibular arch. Prophylactic antibiotics (875 mg Amoxicillin and 125 mg Clavulanic acid) regimen were prescribed to the participants twice daily starting 1 h prior to surgery and continuing for 7 days. Pain was controlled by using non-steroidal anti-inflammatory drug. The participants were instructed to don’t wear the complete denture and eat soft diet.

**Fig. 1:**
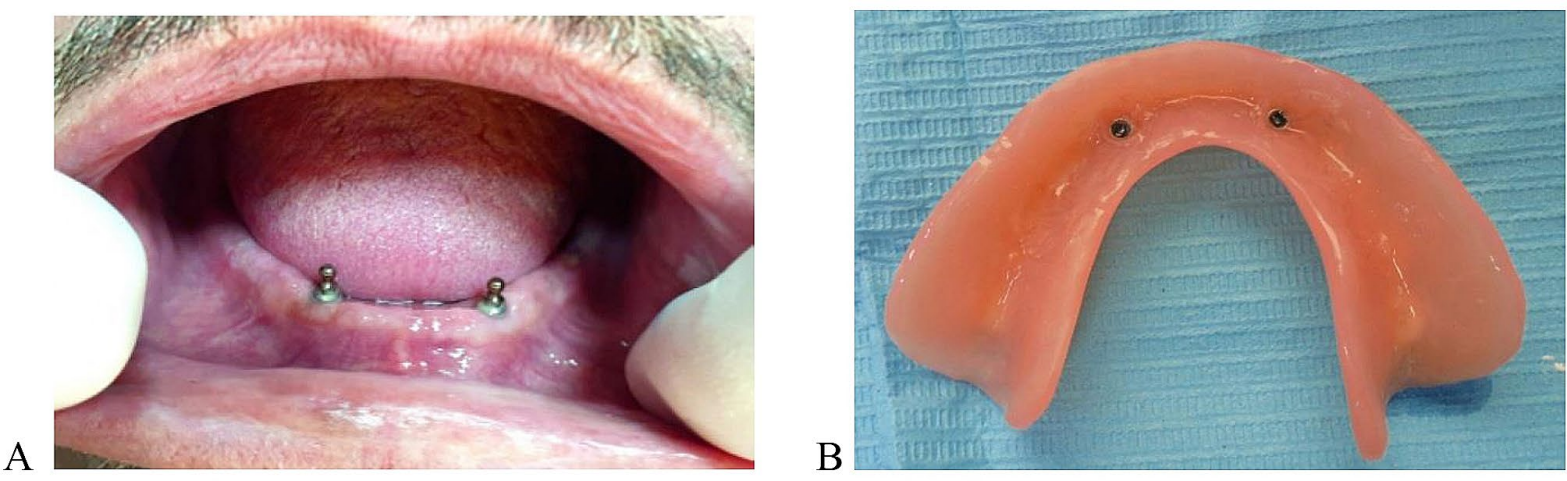
Implant retained overdenture prosthesis. **A,** Ball abutments in patient mouth. **B,** Overdenture fitting surface with the metal housings after pick up procedure

After 10 days, a cold-curing silicone- based relining (Softliner, PROMEDICA) was used to reline the mandibular denture then the denture was delivered to the participant and follow-up appointments were scheduled. After osseointegration period (3–4 months) the dental implants were exposed, the cover screw was removed, and healing abutments were screwed to the dental implants. After 1 week, the healing abutments were unscrewed and ball attachments (Dentium ball abutment, Superline II) were attached into the dental implants (Fig. 1A). The attachment housing was incorporated into the fitting surface of the denture by using direct pick-up approach (Fig. 1B). The occlusion was checked by using articulating paper to remove any occlusal interference and the overdenture was delivered to the patient.

### Videofluroscopic swallowing study (VFSS)

**Fig. 2:**
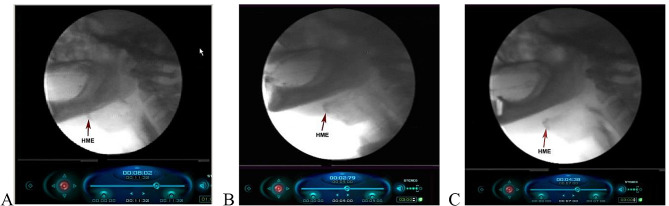
Videofluoroscopy images showing hyoid maximum elevation (HME) for completely edentulous patients at different conditions. **A,** without denture **B,** with complete denture. **C,** with implant overdenture

A videofluoroscopic swallowing study was conducted for each participant at 3 different states: without denture (WCD), 2 months after insertion of the complete dentures (CDs), and 2 months after the insertion of the implant overdentures (IODs). Video fluoroscopic evaluation was done during swallowing of 10 ml thin liquid (80% water and 20% barium sulfate -Prontobario H.D^®^). The recordings data were saved on a computer for future examination. The video frames were timed at 1/100 second and analyzed frame by frame by using a software program (EO Program) (Fig. 2A, B, C).

#### Spatial measurements

According to Rebecca Leonard and Katherine Kendall study, measurements of the superior hyoid displacement (vertical movement) and anterior hyoid displacement (horizontal movement) were taken at both the rest position and during maximum hyoid displacement [26]. To measure this displacement a line was drawn from the anterior-superior point of cervical spine 4 (P1) contacting anterior-inferior point of cervical spine 2 (P2) and extended superiorly if needed.

**Fig. 3:**
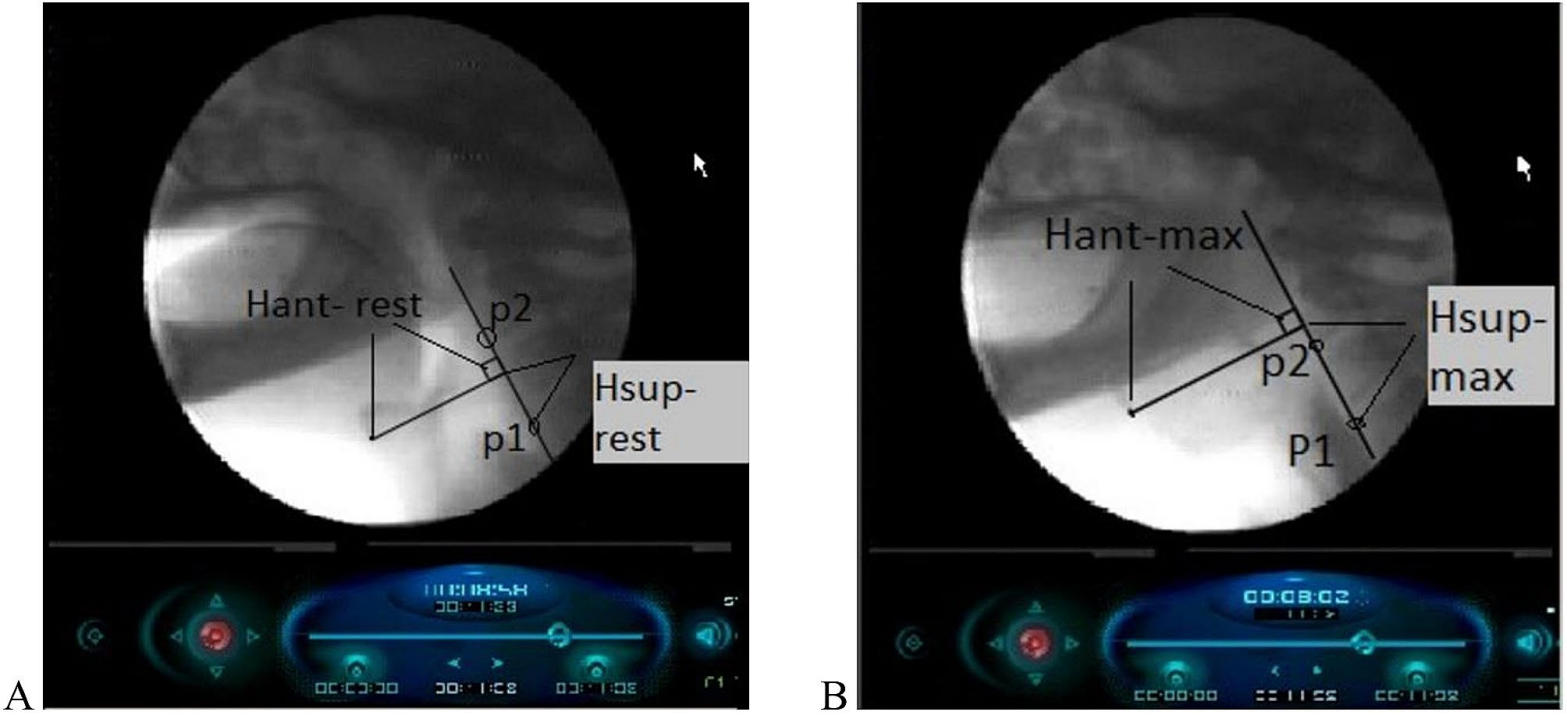
Videofluoroscopy images showing measurement methodology. (**A**) Hyoid bone at rest (**B**) Hyoid bone at maximum elevation

A second line was extended from the anterior-inferior part of the hyoid bone perpendicular to the P1-P2 line. The measurements were taken from the hyoid bone to the intersection point (Hant-rest) and from the P1 to the intersection point (Hsup-rest) (Fig. 3A). The previous procedures was repeated again at the maximum displacement of the hyoid bone. The measurements from the hyoid bone to the intersection point (Hant- max) and from P1 to the intersection point (Hsup-max) were recoded (Fig. 3B). The superior hyoid displacement was referred as the difference between (Hsup-max) and the (Hsup-rest) in the vertical direction however, the anterior hyoid displacement was referred as the difference between (Hant-max) and the (Hant-rest) in the horizontal direction.

#### Temporal measures

*1. Duration of hyoid maximum elevation (DOHME).*

Detected from the first frame displays maximum hyoid elevation to the last frame displays maximum hyoid elevation.

*2. Duration of hyoid maximum anterior excursion (DOHMAE).*

Detected from the first frame displays maximal anterior hyoid movement to the last frame displays the maximum anterior hyoid movement.

#### Penetration / aspiration scale

When food particles enter the airway below the level of the vocal folds, it is referred to as aspiration, whereas penetration is when food particles penetrate the airway down to the vocal folds.

0: No penetration.

1: penetration.

2: Aspiration.

### Statistical analysis

Statistical package for social science version 25 (SPSS Inc.) was used for data analysis. Test of normality was done by using Shapiro-Wilk test. Repeated measures ANOVA with Bonferroni correction of *P* value was used to analyze correlation between different oral conditions. *P* value was considered significant if less than 0.05.

## Results

Table 1 shows the general data of the participants. The mean age of the participants of the study was 60.1 ± 5.82 and they included 15 males and 10 females. The mean duration of edentulism of the participant was 3.6 ± 1.01 and the mean duration of complete denture use was 1.4 ± 1.15. As for socioeconomic status, 15 subjects were low, 8 subjects were medium, and 2 subjects were high socioeconomic status.

**Table 1:** Demographic characteristics of participants (*n* = 25)

Characteristics	No.
Age (years), mean ± SD	60.1 ± 5.82
Gender (male/ female)	15:10
Duration of edentulism (years), mean ± SD	3.6 ± 1.01
Socioeconomic status:	
- Low - Medium - High	15 (60%) 8 (32%) 2 (8%)
Duration of complete denture use (years), mean ± SD	1.4 ± 1.15

**SD**: Standard deviation

**Fig. 4:**
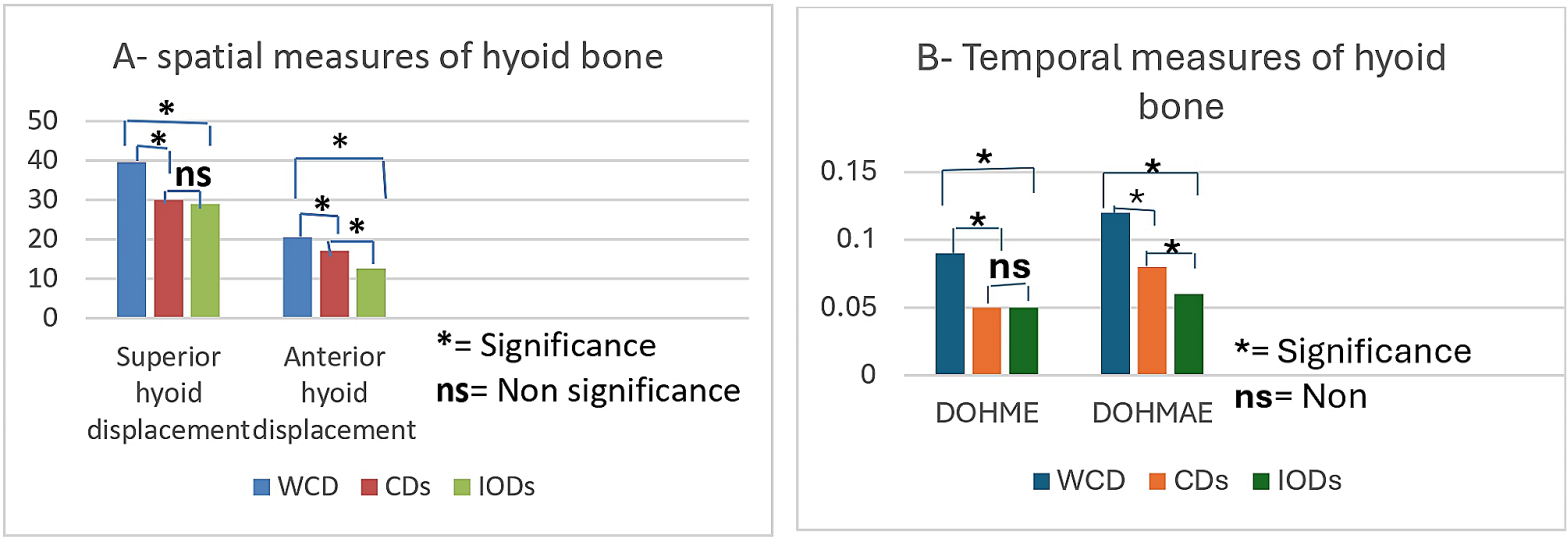
**A, B** Showing videofluroscopic swallowing findings of the hyoid bone for without complete denture, with complete dentures, and with implant overdentures

The descriptive statistics data for the videofluoroscopic swallowing study (VFSS) findings for different oral conditions (WCD, CDs, and IODs) were presented in Table 2; Fig. 4A, B. The highest values were shown for the without denture condition (39.5 ± 5.1, 20.5 ± 3.5, 0.09 ± 0.03, and 0.12 ± 0.02 for superior hyoid displacement, anterior hyoid displacement, DOHME, and DOHMAE, respectively), while the lowest values were found for implant overdentures (29 ± 4.1, 12.5 ± 3.4, 0.05 ± 0.01, and 0.06 ± 0.02 for superior hyoid displacement, anterior hyoid displacement, DOHME, and DOHMAE, respectively).

**Table 2:** Comparison of videofluroscopic swallowing findings (VFSS) between WCD, CDs, and IODs oral conditions

Variables		WCD	CDs	IODs	Comparisons of VFSS findings		
		Mean ± SD	Mean ± SD	Mean ± SD	P1	P2	P3
Spatial measures (mm)	Superior hyoid displacement	39.5 ± 5.1	30 ± 4.3	29 ± 4.1	0.025*	0.015*	0.08
	Anterior hyoid displacement	20.5 ± 3.5	17 ± 3.2	12.5 ± 3.4	0.014*	0.007*	0.034*
Temporal measures (ms)	DOHME	0.09 ± 0.03	0.05 ± 0.02	0.05 ± 0.01	0.015*	0.003*	0.072
	DOHMAE	0.12 ± 0.02	0.08 ± 0.01	0.06 ± 0.02	0.025*	0.002*	0.042*

**WCD**: Without complete denture; **CDs**: Conventional complete dentures; **IODs**: Implant retained overdentures; **SD**: Standard deviation

(Bonferroni test, *P* < 0.05) *P is significant at 5% level

P1: significance between WCD &CDs

P2: significance between WCD& IODs

P3: significance between CDs& IODs

The comparison of the VFSS findings between WCD, CDs, and IODs is presented in Table 2.

### Spatial measurements

Statistically significant reduction in both superior and anterior hyoid displacements were found for conventional dentures and implant overdentures when compared to the without denture oral condition (*P* < 0.05). Additionally, there was a statistically significant decrease in anterior hyoid displacement for implant overdentures compared to the conventional complete dentures (*P* < 0.05), while there was no significant difference was found in superior hyoid displacement between the two oral conditions (*P* > 0.05).

### Temporal measurements

There was a statistically significant increase in the duration of hyoid maximum elevation and the duration of the hyoid maximum anterior excursion for WCD compared to the CDs or IODs (*P* < 0.05). Implant retained overdentures showed a statistically significant reduction in the duration of hyoid maximum anterior excursion compared to conventional complete dentures (*P* < 0.05), while there was no significant difference in the duration of hyoid maximum elevation between the two oral conditions (*P* > 0.05).

No aspiration was found in any of the three different oral conditions. However, penetration of food bolus during swallowing was found in the without denture condition for the majority of patients {23 patients (92%)}. After wearing conventional complete dentures or implant overdentures, there was no penetration of food bolus.

## Discussion

During the swallowing process of edentulous patient, the pharyngeal area is expanded due to the superior rotation of the mandible and forward pulling of the tongue [27]. These changes are compensated for by an increase in the displacement of the hyoid bone and larynx [12, 28]. The present study showed a significant reduction in both superior and anterior hyoid displacement for conventional complete dentures and for implant overdentures compared to the state without denture. This result rejects the null hypothesis. These findings are similar to a study by Onodera et al., which reported an increase in hyoid displacement when swallowing without complete dentures. Additionally, the fixation of the mandible through occlusal contacts is crucial for hyoid elevation, which initiates the pharyngeal swallowing [12].

Hattori (2004) found that the vertical dimension of occlusion has a strong influence on hyoid bone displacement during swallowing [28]. In this study, there was a significant reduction in temporal measures of hyoid bone displacement (DOHME and DOHMAE) for both implant overdentures and conventional dentures compared to the state without dentures. This may be due to changes in the vertical dimension of occlusion after the insertion of conventional dentures or an implant overdenture. This findings is supported by Takagi et al. (2021), who found that wearing dentures decreases the pharyngeal transit time [13].

Mandibular implant overdentures were associated with a reduction in anterior hyoid displacement and DOHMAE compared to conventional complete dentures. The placement of dental implants enhances the retention and stability of the implant overdentures, resulting in more comfortable and more stable tongue movement, which can affect swallowing function. Ellis et al., reported that the mandible act as a support point to move the hyoid bone and triggering the transfer of the food bolus to the stomach. However, during swallowing with the complete dentures, the potential movement of the mandible was associated with slight movement of the denture on the bearing mucosa, reducing the functional efficiency of the conventional complete dentures [29]. Monaco et al., also reported that increasing denture retention leads to a reduction in total swallow duration [14]. Another explanation for the reduced anterior hyoid displacement in implant overdentures compared to conventional complete dentures may be attributed to the restoration of sensory and motor feedback through Osseo perception [30]. As swallowing is a sensorimotor behavior [20, 31, 32], the placement of dental implants may improve the placement of the mandible during swallowing through new sensory feedback.

The lack of difference in both superior hyoid displacement and DOHME between implant overdentures and conventional complete dentures may be attributed to the fact that vertical displacement of the hyoid bone was more related to changes in vertical dimension rather than tongue movement during swallowing, which is maintained after complete dentures or implant overdentures insertion. This findings is in agreement with Ishida et al., who reported that the vertical displacement of the hyoid bone was highly variable and related to the oral processes only [33].

This study found that, laryngeal penetration during swallowing was found most commonly observed in the edentulous patients not wearing conventional complete dentures or implant overdentures. This may be due to the coordination of swallowing movements between the tongue, hyoid bone, and the larynx being disrupted by the loss of occlusal support during swallowing. This finding is similar to a study by Yoshikawa et al., which found that the incidence of laryngeal penetration during swallowing was commonly in completely edentulous patients not wearing dental prostheses [34].

The within-subject study design used in this study allows for the standardization of several factors that could affect the measurement of hyoid displacement, such as age, sex, and muscular activity. Additionally, the displacement of the hyoid bone may be influenced by the volume and texture of the swallowed food bolus [35, 36]. Therefore, a standardized 10 ml thin liquid bolus was used in this study to establish basic data and simplify the evaluation of results. The limitations of the present study include the small sample size and the test food was limited to thin liquid bolus only. A sequential evaluation from mastication to swallowing is necessary to evaluate the effect of implant overdenture. Accordingly, future researches with large patient samples and with different food bolus consistencies are needed.

## Conclusions

According to the results of this study, mandibular implant-retained overdentures have a positive effect on hyoid displacement during swallowing process for thin liquid bolus consistency. Therefore, the rehabilitation of completely edentulous patients can be performed more effectively using mandibular implant overdentures, not only to improve oral masticatory function, but also pharyngeal swallowing.

### Electronic supplementary material

Below is the link to the electronic supplementary material.

Supplementary Material 1

Supplementary Material 2

Supplementary Material 3

Supplementary Material 4

## Data Availability

The datasets used and analyzed for the current study are available from the corresponding author on reasonable request.

## Abbreviations

WCD: Without complete dentures
CDs: Conventional complete dentures
IODs: Implant retained overdentures
Hant-rest: Hyoid anterior rest
Hsup-rest: Hyoid superior rest
Hant-: Max Hyoid anterior maximum
Hsup-max: Hyoid superior maximum
HME: Hyoid maximum elevation
