# Supplementary material for: Hyoid displacement during swallowing function for completely edentulous subjects rehabilitated with mandibular implant retained overdenture
Source: BMC Oral Health. 2024 Aug 8;24:914. doi: 10.1186/s12903-024-04616-9 (PMC11312938; doi:10.1186/s12903-024-04616-9)
Supplement: Supplementary file 1 — Supplementary Material 1 [file 12903_2024_4616_MOESM1_ESM.docx]

**Justification letter**

**Details of the authors:**

**1- Abdallah Mohammed Ibrahim BDS, MSc, Ph.D.** (Corresponding author)

Associate professor, Department of Removable prosthodontics, Faculty of Dentistry, Mansoura University, Eldakahlia, Egypt.

*E-mail:* [abdallahs@mans.edu.eg](mailto:abdallahs@mans.edu.eg)

**Role during the research:** Planning for study design, help in writing and collection of data.

**2- Mohamed Elgamal BDS, MSc, PhD.**

Associate professor, Department of Removable prosthodontics, Faculty of Dentistry, Mansoura University, Eldakahlia, Egypt and Associate professor in the department of Removable Prosthodontics, Faculty of Dentistry, Horus University, Damietta, Egypt.

*E-mail:* [drelgamal@mans.edu.eg](mailto:drelgamal@mans.edu.eg)

**Role during the research:** Placement of superstructure prostheses and analysis of collected data.

**3- Elsayed Abdallah Abdel-Khalek BDS, MSc, Ph.D.**

Associate professor in the department of Removable Prosthodontics, Faculty of Dentistry, Mansoura University.

E-mail: elsayed_abdallah@mans.edu.eg

**Role during the research**: Help in writing and collection of data.
