# Supplementary material for: Hyoid displacement during swallowing function for completely edentulous subjects rehabilitated with mandibular implant retained overdenture
Source: BMC Oral Health. 2024 Aug 8;24:914. doi: 10.1186/s12903-024-04616-9 (PMC11312938; doi:10.1186/s12903-024-04616-9)
Supplement: Supplementary file 3 — Supplementary Material 3 [file 12903_2024_4616_MOESM3_ESM.docx]

**Hyoid displacement during swallowing function for completely edentulous subjects rehabilitated with mandibular implant retained overdenture.**

**Abdallah Mohammed Ibrahim BDS, MSc, PhD,^a*^ Mohamed Elgamal BDS, MSc, PhD,^b^ Elsayed Abdallah Abdel-Khalek BDS, MSc, PhD.^c^**

**Declarations of interest: none.**

^a^Associate professor, Department of Removable prosthodontics, Faculty of Dentistry, Mansoura University, Eldakahlia, Egypt.

^b^Associate professor, Department of Removable prosthodontics, Faculty of Dentistry, Mansoura University, Eldakahlia, Egypt and Associate professor in the department of Removable Prosthodontics, Faculty of Dentistry, Horus University, Damietta, Egypt.

^c^Associate professor, Department of Removable prosthodontics, Faculty of Dentistry, Mansoura University, Eldakahlia, Egypt.

***Corresponding Author**

*Abdallah Mohammed Ibrahim,*

*Associate professor, Department of Removable Prosthodontics, Faculty of Dentistry,*

*Mansoura University, Eldakahlia, Egypt.*

*P.O.Box:35516*

*#68 ElGomhoria Street, ElMansoura*

*Phone: 00201003565380*

*Fax: +502260173*

*E-mail:* [abdallahs@mans.edu.eg](mailto:abdallahs@mans.edu.eg)
