# Supplementary material for: Hyoid displacement during swallowing function for completely edentulous subjects rehabilitated with mandibular implant retained overdenture
Source: BMC Oral Health. 2024 Aug 8;24:914. doi: 10.1186/s12903-024-04616-9 (PMC11312938; doi:10.1186/s12903-024-04616-9)
Supplement: Supplementary file 4 — Supplementary Material 4 [file 12903_2024_4616_MOESM4_ESM.pdf]

ClinicalTrials.gov Protocol Registration and Results System (PRS) Receipt
Release Date: February 26, 2024

ClinicalTrials.gov ID: NCT06187181

Study Identification

Unique Protocol ID: A19061222

Brief Title: Hyoid Displacement During Swallowing Function for Completely Edentulous Subjects

Official Title: Hyoid Displacement During Swallowing Function for Completely Edentulous Subjects Rehabilitated With Mandibular Implant Retained Overdenture

Secondary IDs:

Study Status

Record Verification: December 2023

Overall Status: Completed

Study Start: December 6, 2022 [Actual]

Primary Completion: July 10, 2023 [Actual]

Study Completion: November 20, 2023 [Actual]

Sponsor/Collaborators

Sponsor: Mansoura University

Responsible Party: Sponsor

Collaborators:

Oversight

U.S. FDA-regulated Drug: No

U.S. FDA-regulated Device: No

U.S. FDA IND/IDE: No

Human Subjects Review: Board Status: Approved
Approval Number: A19061222
Board Name: Dental Research Ethics Committee
Board Affiliation: DREC
Phone: 01003565380
Email: abdallahs79@hotmail.com
Address:

#68 ElGomhoria Street, ElMansoura

Data Monitoring:

Study Description

Brief Summary: To investigate the effect of wearing mandibular implant retained overdentures on superior and anterior hyoid displacement during swallowing process.

Detailed Description: New conventional complete denture with optimal flange extension and well established lingualized balanced occlusal scheme was constructed for every patient. To construct the radiographic stent, clear acrylic resin duplicate for the mandibular denture was constructed, several small holes were made on the polished surface of the duplicate. These holes were filled with radio-opaque gutta-percha points to act as a reference points during determination of the quantity of available bone for implant placement and for construction of stereolithographic surgical guide.

Cone beam computerized tomography [i-CAT] was used to construct the stereolithographic surgical guide by applying dual scan technique. By using the surgical guide, two dental implants (Dentium implants, 12–14mm in length, 3.6-4mm in diameter, Superline II) were placed in the canine regions of the mandibular arch with guided surgical protocol. The patients were instructed to take prophylactic antibiotic two times a day (875mg Amoxicillin and 125mg Clavulanic acid) 24 hours before the surgery and continue after the surgery for seven days. Non-steroidal anti-inflammatory drug was used to control the pain.

After surgery, the complete denture was withdrawn, and the patient was instructed to eat soft diet. After one week, the mandibular denture was relieved and relined with Cold- curing silicon based relining material (Softliner, PROMEDICA). The dentures were delivered to the patients and follow up visits were scheduled. After three to four months, the dental implants were exposed, and the healing abutments were secured to the dental implants. After healing of the soft tissue (7-10 days), the healing abutments were removed, and ball attachments (Dentium ball abutment, Superline II) were screwed to the dental implants. Direct pick-up technique was used to incorporate the housing of the attachments into the fitting surface of the mandibular denture using self-cured acrylic resin material. The occlusion of the overdenture was evaluated to eliminate any premature occlusal contact then the overdenture was delivered to the patient.

Videofluroscopic swallowing study (VFSS):

All subjects were examined by using videofluroscopic swallowing study to evaluate bolus movements. This evaluation was done at three different oral conditions, without complete denture (WCD), after 2 months of conventional complete dentures insertion (CDs), and after 2 months of implant overdenture insertion (IODs). Each patient was instructed to swallow 10 ml of thin liquid {20% barium sulfate (Prontobario H.D®) and 80% water} three times. The records of the videofluroscopic swallowing evaluation were saved to the computer for later analysis. A software Program (EO Program) was used to placing timing on the frame of the video (1/100 second) and analyze the recording by frame by frame data analysis.

a- Spatial measurements: Superior hyoid displacement (vertical movement) and anterior hyoid displacement (horizontal movement) were measured according to dynamic swallowing study performed by Rebecca Leonard & Katherine Kendall during pseudo rest position and during maximum overall hyoid displacement .24 Aline was drawn from anterior superior point of cervical spine 4 (p1) contacting anterior inferior point of cervical spine 2 (p2) and extended superiorly as necessary, then another line was extended from the anterior, inferior portion of the hyoid bone to meet the P1-P2 line at 90-degree intersection. The Measurement from the P1 to the intersection point (Hsup-rest) and from hyoid bone to the intersection point (Hant-rest) were recorded then the video was advanced until the hyoid bone showed maximum overall displacement and

the previous procedure was repeated. The measurements from P1 to the intersection point (Hsup-max) and from the hyoid bone to the intersection point (Hant- max) were recoded. The difference between the maximum hyoid position (Hsup-max) and the pseudo rest position (Hsup-rest) in the vertical direction was called the superior hyoid displacement, while the difference between maximum hyoid position (Hant-max) and pseudo rest position (Hant-rest) in the horizontal direction was called anterior hyoid displacements.

B- Temporal measurements:

1. Duration of hyoid maximum elevation (DOHME):

   Measured from the first frame showing maximum hyoid elevation to last frame showing maximum hyoid elevation.
2. Duration of hyoid maximum anterior excursion (DOHMAE):

Measured from the first frame showing maximum anterior hyoid movement to last frame showing maximum anterior hyoid movement.

C- Penetration / aspiration scale:

Penetration was defined as the food bolus enter the airway down to the vocal folds, while aspiration defined as the food bolus enter the airway below the level of the vocal folds.

0= No penetration or aspiration

1. Penetration
2. Aspiration

Conditions

Conditions: Prosthesis Durability

Keywords: Hyoid bone.
implant.
overdenture
swallowing.

Study Design

Study Type: Interventional

Primary Purpose: Treatment

Study Phase: N/A

Interventional Study Model: Crossover Assignment

Number of Arms: 3

Masking: Single (Participant)

Allocation: Non-Randomized

Enrollment: 25 [Actual]

Arms and Interventions

Arms	Assigned Interventions
Placebo Comparator: G. I : Completely edentulous patients<br>videofluroscopy for completely edentulous patients	Radiation: videofluroscopy<br>videofluroscopy evaluation of swallowing function

Arms	Assigned Interventions
Active Comparator: G.II: patients with complete dentures<br>complete denture construction for edentulous patient and videofluroscopy evaluation	Procedure/Surgery: implant overdenture<br>. Videofluoroscopy swallowing evaluation was done using 10 ml of thin liquid bolus at three different oral conditions: Without dentures (WOD), with the conventional complete denture (CDs) and with mandibular implant retained overdenture (IODs).<br>Radiation: videofluroscopy<br>videofluroscopy evaluation of swallowing function
Active Comparator: G.III: patients with implant overdentures<br>construction of implant overdenture and videofluroscopy evaluation	Procedure/Surgery: implant overdenture<br>. Videofluoroscopy swallowing evaluation was done using 10 ml of thin liquid bolus at three different oral conditions: Without dentures (WOD), with the conventional complete denture (CDs) and with mandibular implant retained overdenture (IODs).<br>Radiation: videofluroscopy<br>videofluroscopy evaluation of swallowing function

Outcome Measures

Primary Outcome Measure:

1. swallowing evaluation
   hyoid bone displacement evaluation by videofluriscopy

   [Time Frame: after 3 months of denture or overdenture insertion]

Eligibility

Minimum Age: 50 Years

Maximum Age: 70 Years

Sex: All

Gender Based: No

Accepts Healthy Volunteers: Yes

Criteria: Inclusion Criteria:

- free from any medical problems that may affect the osseointegration of the dental implants
- class I maxillomandibular relationship and free from any previous swallowing problems

Exclusion Criteria:

- participants with temporomandibular joint disorder,
- head and neck surgery,
- sleep apnea
- skeletal deformity

Contacts/Locations

Central Contact Person: Abdallah Ibrahim, PhD
Telephone: 01003565380
Email: abdallahs79@hotmail.com

Central Contact Backup:

Study Officials: abdallah Ibrahim, PhD
Study Principal Investigator
Mansoura university, Faculty of dentistry, prosthodontics department

Locations: **Egypt**
faculty of dentistry, mansoura university
Mansoura, Egypt, 35516
Contact: abdallah Ibrahim, PhD 01003565380 abdallahs79@hotmail.com

IPDSharing

Plan to Share IPD: No

References

Citations: Kern JS, Kern T, Wolfart S, Heussen N. A systematic review and meta-analysis of removable and fixed implant-supported prostheses in edentulous jaws: post-loading implant loss. Clin Oral Implants Res. 2016 Feb;27(2):174-95. doi: 10.1111/clr.12531. Epub 2015 Feb 9. PubMed 25664612

Links:

Available IPD/Information:

U.S. National Library of Medicine | U.S. National Institutes of Health | U.S. Department of Health & Human Services
